# Disordered GPR43/NLRP3 expression in peripheral leukocytes of patients with atrial fibrillation is associated with intestinal short chain fatty acids levels

**DOI:** 10.1186/s40001-024-01825-4

**Published:** 2024-04-15

**Authors:** Chen Fang, Kun Zuo, Zheng Liu, Li Xu, Xinchun Yang

**Affiliations:** 1 Department of Cardiology, Nanjing Drum Tower Hospital, The Affiliated Hospital of Nanjing University Medical School, Nanjing University, No. 321 Zhongshan Road, Nanjing, 210008 China; 2 Beijing Key Laboratory of Hypertension, Beijing Chaoyang Hospital, Heart Center, Capital Medical University, 8th Gongtinanlu Rd, Chaoyang District, Beijing, 100020 China

**Keywords:** Atrial fibrillation, Peripheral leukocytes, GPR43, NLRP3, SCFAs

## Abstract

**Background:**

Atrial fibrillation (AF) is associated with circulating inflammation. Short-chain fatty acids (SCFAs) derived from gut microbiota (GM) regulate leukocyte function and inhibit the release of inflammatory cytokines, which are partly mediated by the G-protein-coupled receptor 43 (GPR43) signaling. This study aimed to investigate the expression of GPR43/NOD-like receptors family pyrin domain containing 3 (NLRP3) in leukocytes and the interaction with intestinal SCFAs levels in AF patients.

**Methods:**

Expressions of GPR43 and NLRP3 mRNA in peripheral blood leukocytes from 23 AF patients and 25 non-AF controls were detected by quantitative reverse transcription-polymerase chain reaction (qRT-PCR). Expressions of leukocyte GPR43 and NLRP3 protein were evaluated by western blot analysis. The levels of plasma IL-1β were measured by enzyme-linked immunosorbent assay (ELISA). The fecal SCFAs levels based on GC/MS metabolome of corresponding 21 controls and 14 AF patients were acquired from our published dataset. To evaluate the expression of NLRP3 and GPR43 and the release of IL-1β, human THP-1 cells were stimulated with or without SCFAs (acetate, propionate, and butyrate), lipopolysaccharide (LPS), and nigericin in vitro, respectively.

**Results:**

Compared to the controls, the mRNA expression in peripheral leukocytes was significantly reduced in AF patients (*P* = 0.011) coupled with the increase in downstream leukocyte NLRP3 mRNA expression (*P* = 0.007) and plasma IL-1β levels (*P* < 0.001), consistent with changes in GPR43 and NLRP3 protein expression. Furthermore, leukocyte GPR43 mRNA levels were positively correlated with fecal GM-derived acetic acid (*P* = 0.046) and negatively correlated with NLRP3 mRNA expression (*P* = 0.024). In contrast to the negative correlation between left atrial diameter (LAD) and GPR43 (*P* = 0.008), LAD was positively correlated with the leukocyte NLRP3 mRNA levels (*P* = 0.024). Subsequent mediation analysis showed that 68.88% of the total effect of intestinal acetic acid on AF might be mediated by leukocyte GPR43/NLRP3. The constructed GPR43–NLRP3 score might have a predictive potential for AF detection (AUC = 0.81, *P* < 0.001). Moreover, SCFAs treatment increased GPR43 expression and remarkably reduced LPS/nigericin-induced NLRP3 expression and IL-1β release in human THP-1 cells in vitro.

**Conclusions:**

Disrupted interactions between GPR43 and NLRP3 expression in peripheral blood leukocytes, associated with reduced intestinal GM-derived SCFAs, especially acetic acid, may be involved in AF development and left atrial enlargement by enhancing circulating inflammation.

**Supplementary Information:**

The online version contains supplementary material available at 10.1186/s40001-024-01825-4.

## Background

Atrial fibrillation (AF) is the most common cardiac arrhythmia in clinical practice, independently associated with substantial morbidity and a 1.5- to fourfold increased risk of mortality [1–3], and has been associated with inflammation in the systemic circulation, particularly immune cells and cytokines that mediate inflammatory responses [3–5]. A greater understanding of the complex mechanisms of AF-related inflammation may facilitate anti-inflammatory strategies to prevent AF.

Recent evidence suggests that gut microbiota (GM) homeostasis protects host immune responses and inflammatory processes by generating bioactive metabolites [6, 7]. Short-chain fatty acids (SCFAs), the important GM-derived metabolites produced by the fermentation of dietary fiber in the colon and the distal small intestine, absorb into the circulation and bind to the G-protein-coupled receptor 43 (GPR43), profoundly regulating inflammatory responses [8, 9]. Animal studies indicate that stimulation of GPR43 by SCFAs is necessary to alleviate inflammation and that GPR43-deficient immune cells, especially leukocytes, increase the production of inflammatory mediators and the recruitment of immune cells [7]. Moreover, SCFAs could inhibit the activation of the NOD-like receptor family, pyrin domain containing 3 (NLRP3) inflammasome, which is expressed on leukocytes and is critical for the release of pro-inflammatory mediators [10–14]. Notably, systemic inflammation mediated by pro-inflammatory cytokines contributes to left atrial remodeling, marked by left atrial enlargement, involving AF pathophysiology [15–17]. Our previous studies confirmed the disordered GM and decreased SCFAs in the gut of AF patients, as well as dysregulation of the cardiac SCFA–GPR43–NLRP3 interactions during AF development and atrial remodeling [18–20]. However, the relationship between intestinal GM-derived SCFAs, leukocyte GPR43 and NLRP3 expression, and AF remains unclear.

Thereby, this study aimed to investigate the role of leukocyte GPR43/NLRP3 expression in AF patients and whether it is linked to GM-derived SCFAs.

## Methods

### Study population

A total of 48 subjects with (*n* = 23) and without AF (*n* = 25) from Beijing Chaoyang Hospital Affiliated to Capital Medical University were recruited for this study. The diagnostic criteria for AF according to 2020 European Society of Cardiology (ESC) guidelines [2]. The exclusion criteria included structural heart disease, coronary heart disease, heart failure, metabolic syndrome, irritable bowel syndrome, acute or chronic infection, autoimmune disease, liver disease, renal disease, cancer, and use of probiotics, antibiotics, steroids, or proton pump inhibitors in the past 1 month. Clinical characteristics of subjects, including age, gender, medical history, etc., were collected at the time of enrollment. Echocardiography was performed on corresponding 21 controls without AF and 23 AF patients, and results were recorded. The research protocol conformed to the Declaration of Helsinki and was approved by the ethics committee of Beijing Chaoyang Hospital Affiliated to Capital Medical University (Ethical approval number: 2019-KE-57). All the participants signed informed consent forms.

### Expression analysis on GPR43 and NLRP3

Peripheral blood samples were collected from an antecubital vein during the fasting state in the morning. After centrifugation at 4 °C and 3000 rpm for 10 min, plasma was separated and stored for the follow-up detection. Then peripheral blood leukocytes were isolated, and cell RNA extraction was performed using RNA Extraction Kit (Servicebio, Wuhan, China). Nano drop was used to detect extracted RNA concentration. Following cDNA synthesis, Real-Time Quantitative Reverse Transcriptase-Polymerase Chain Reaction Analysis (qRT-PCR) was performed to examine the expression of GPR43 and NLRP3 using CFX96 Q-PCR system (Bio-rad, USA). GAPDH was used as the internal control. The sequences of the used primers are listed in Table 1.

**Table 1:** Sequence primers used for qRT-PCR

	Forward	Reverse
GPR43	5′-GCAGGGGCTAAAGCTCTGTT-3′	5′-TGTTGTTCCGGATAGGTGGC-3′
NLRP3	5′-AGCACCAGCCAGAGTCTAAC-3′	5-CCCCAACCACAATCTCCGAAT-3′
GAPDH	5′-GGAAGCTTGTCATCAATGGAAATC-3′	5′-TGATGACCCTTTTGGCTCCC-3′

*GPR43* G-protein-coupled receptor 43, *NLRP3* NOD-like receptor family, pyrin domain containing 3

### Fecal SCFAs quantification based on GC–MS

In the present cohort, corresponding 21 non-AF controls and 14 AF patients underwent GC–MS-based fecal SCFAs quantification and the dataset was available from our previous published study [18].

### Cell culture and stimulation

Human THP-1 cells purchased from Pricella (CL-0233) were cultured in RPMI-1640 medium supplemented with 10% fetal bovine serum (Gibco) and 1% penicillin–streptomycin (Gibco) at 37 °C in a 5% CO_2_ incubator. After stimulation with or without SCFAs, including sodium propionate (0.5 mM), sodium butyrate (0.5 mM), and sodium acetate (10 mM) (Sigma-Aldrich) for 2 h, the cells were treated with LPS (500 ng/mL) for 4 h and then nigericin (10 μM) for 1 h. Following stimulation, culture supernatants and cellular lysates were collected.

### Measurement of IL-1β

The levels of IL-1β in plasma and cellular supernatant were detected with a commercialized human IL-1β ELISA Kit (Proteintech, KE00021). The operation procedures followed the manufacturer’s protocol.

### Western blotting

After determining the extractive total protein concentration of isolated peripheral blood leukocytes and human THP-1 cells using a BCA protein assay kit (Thermo Fisher Inc.), the equivalent amount of 20 μg protein was separated by 10% SDS-PAGE and then transferred to a PVDF membrane. Subsequently, the membranes were blocked with 5% non-fat milk for 1 h at room temperature and incubated with primary antibodies against GPR43, NLRP3, GAPDH, and β-tubulin at 4 °C overnight. GAPDH and β-tubulin were used as endogenous control. Antibodies were obtained from Cell Signaling Technology, Proteintech, and Bioss. Following incubation with secondary antibodies at ambient temperature for 1 h, the immunoreactive bands were visualized and then analyzed by Image J software.

### Statistical analysis

Continuous variables in normal distribution were present as the mean ± SD and analyzed using Student’s *t*-test. Continuous variables in non-normal distribution were expressed as median (quartile) and analyzed using Mann–Whitney test. Categorical variables were presented as numbers (percentages) and analyzed using Fisher’s exact test. Spearman’s correlation was calculated to evaluate the relationship between GPR43, NLRP3, SCFAs, IL-β and clinical features. ROC curve analysis was used to assess the predictive value of the variables. Partial least squares structural equation modeling (PLS-SEM) was applied to evaluate the mediating effects. All statistical analyses were performed using SPSS version 23 (IBM Corp), R-Studio version 1.4.1103 (The R Development Core Team, Vienna, Austria), SmartPLS version 3 (SmartPLS GmbH, Germany) and MedCalc version 20.021 (MedCalc Software Ltd, Ostend, Belgium). *P* values less than 0.05 were considered statistically significant.

## Results

### Basic characteristics

Overall, 25 non-AF controls (age 63.12 ± 9.52 years) and 23 AF patients (age 61.04 ± 9.04 years) were included in the current study. There were no significant differences in age, gender, body mass index (BMI), hypertension (HTN), diabetes mellitus (DM), total cholesterol (TC), triglyceride (TG), alanine aminotransferase (ALT), aspartate aminotransferase (AST), and serum creatinine (sCr) between the two groups. The specific clinical features are presented in Table 2.

**Table 2:** Baseline clinical characteristics of the participants with or without AF

	Control	AF	*P* value
Number	25	23	
Age, years	63.12 ± 9.52	61.04 ± 9.04	0.443
Male (%)	15 (60.00%)	10 (43.48%)	0.386
BMI, kg/m^2^	26.48 ± 3.03	26.97 ± 4.36	0.725
HTN (%)	21 (84.00%)	13 (56.52%)	0.057
DM (%)	5 (20.00%)	3 (13.04%)	0.703
TC, mmol/L	4.25 ± 0.88	4.24 ± 0.90	0.958
TG, mmol/L	1.19 (0.91, 1.98)	1.34 (0.98, 1.95)	0.733
AST, U/L	17.89 ± 7.01	22.96 ± 10.76	0.058
ALT, U/L	17.68 ± 10.22	23.13 ± 12.16	0.098
sCr, μmol/L	69.01 ± 12.54	68.03 ± 12.37	0.785
LAD, mm	36.86 ± 4.49	42.57 ± 6.56	0.002

Data are presented as mean ± SD, median (quartile) or number (%)

*ALT* alanine aminotransferase, *AST* aspartate aminotransferase, *BMI* body mass index, *DM* diabetes mellitus, *HTN* hypertension, *LAD* left atrial diameter, *sCr* serum creatinine, *TC* total cholesterol, *TG* triglyceride

### Altered leukocyte GPR43 and NLRP3 expression in AF patients

**Fig. 1:**
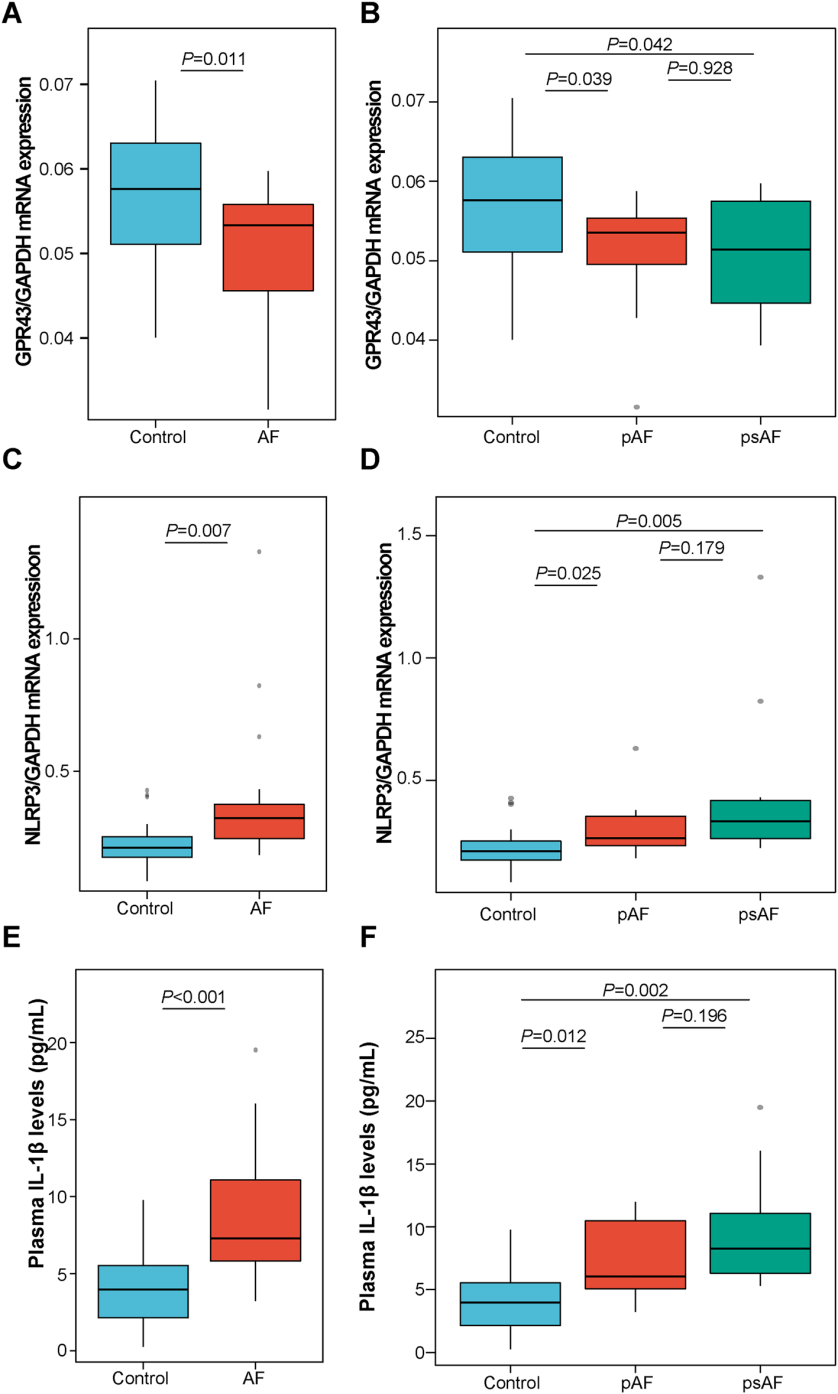
GPR43 and NLRP3 mRNA expression in peripheral blood leukocytes and plasma IL-1β levels between participants with and without AF. **A** Significantly decreased expression of leukocyte GPR43 mRNA in AF patients. *T*-test. **B** A decreasing trend of leukocyte GPR43 mRNA expression in patients with paroxysmal and persistent AF. *T*-test. **C** Significantly increased expression of leukocyte NLRP3 mRNA in AF patients. *T*-test. **D** The expression of NLRP3 mRNA in leukocytes of patients with paroxysmal and persistent AF increased gradually. *T*-test. **E** Plasma IL-1β levels elevated significantly elevated in AF patients. *T*-test. **F** An increasing trend of plasma IL-1β levels in patients with paroxysmal and persistent AF. *T*-test

**Fig. 2:**
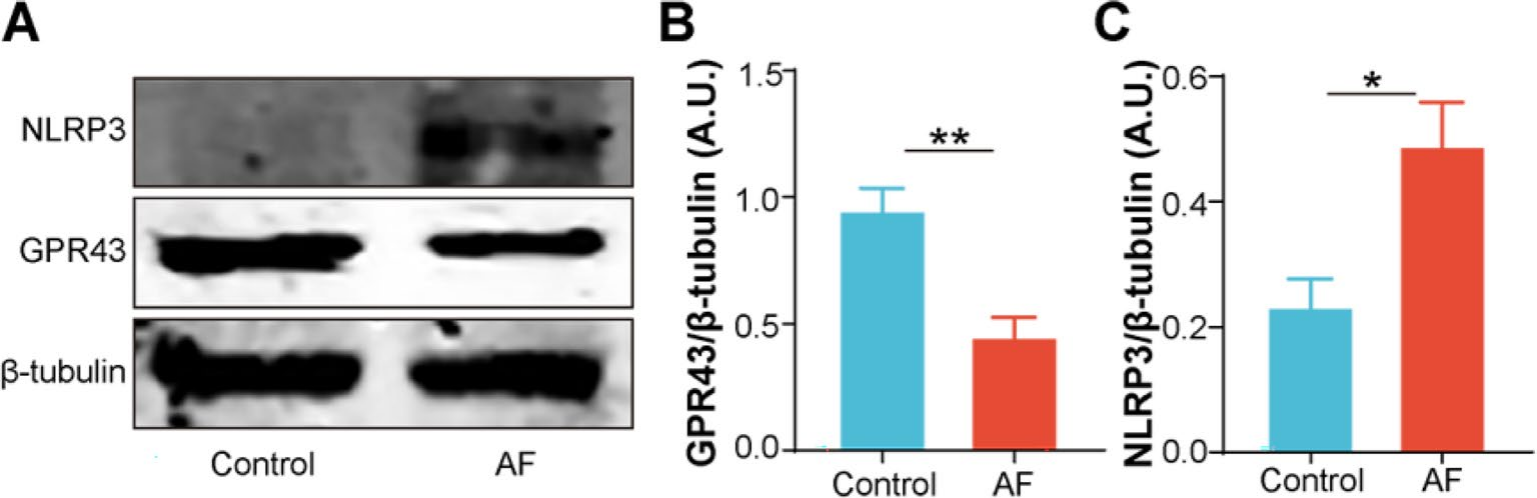
The leukocyte GPR43 and NLRP3 protein expression in participants with and without AF. **A**–**C** Western blotting showed markedly decreased GPR43 protein expression and increased NLRP3 protein expression in AF patients. β-Tubulin was utilized as an endogenous control. A.U., arbitrary units; *n* = 6–7 for each group; *P < 0.05; **P < 0.01; data are mean ± SEM; *T*-test

In peripheral blood leukocytes, the GPR43 mRNA expression was significantly reduced (0.05 ± 0.01 vs. 0.06 ± 0.01, *P* = 0.011) and the NLRP3 was remarkably increased (0.38 ± 0.25 vs. 0.23 ± 0.09, *P* = 0.007) in AF patients compared to the controls based on qRT-PCR analyses (Fig. 1A, C), consistent with the alterations of GPR43 and NLRP3 protein expression (Fig. 2A–C). The expression of leukocyte GPR43 mRNA in patients with paroxysmal AF (pAF) and persistent AF (psAF) represented a decreasing trend, while NLRP3 was gradually increased (Fig. 1B, D). Although the difference between the pAF and psAF groups was not statistically significant, possibly caused by the limited sample size, these changes initially indicated that the abnormal expression of GPR43 and NLRP3 in leukocytes might be involved in AF development.

### Increased plasma IL-1β levels in AF patients

Plasma IL-1β levels in corresponding 24 non-AF controls and 21 AF patients from this study cohort were measured by an ELISA assay, finding that significantly higher IL-1β levels in AF patients than in controls (AF patients vs. Controls, 8.53 ± 4.10 vs. 3.95 ± 2.42 pg/mL; *P* < 0.001) (Fig. 1E). Meanwhile, circulating IL-1β levels exerted an increasing trend in pAF and psAF patients (Fig. 1F). IL-1β, as a product of NLRP3 activation in myeloid cells (including monocytes, neutrophils, and eosinophils) and the core of inflammatory response [21–23], is positively correlated with leukocyte NLRP3 mRNA levels (*R* = 0.40, *P* = 0.007) and AF occurrence (*R* = 0.61, *P* < 0.001).

### Correlation between SCFAs, GPR43 and NLRP3 in AF patients

**Fig. 3:**
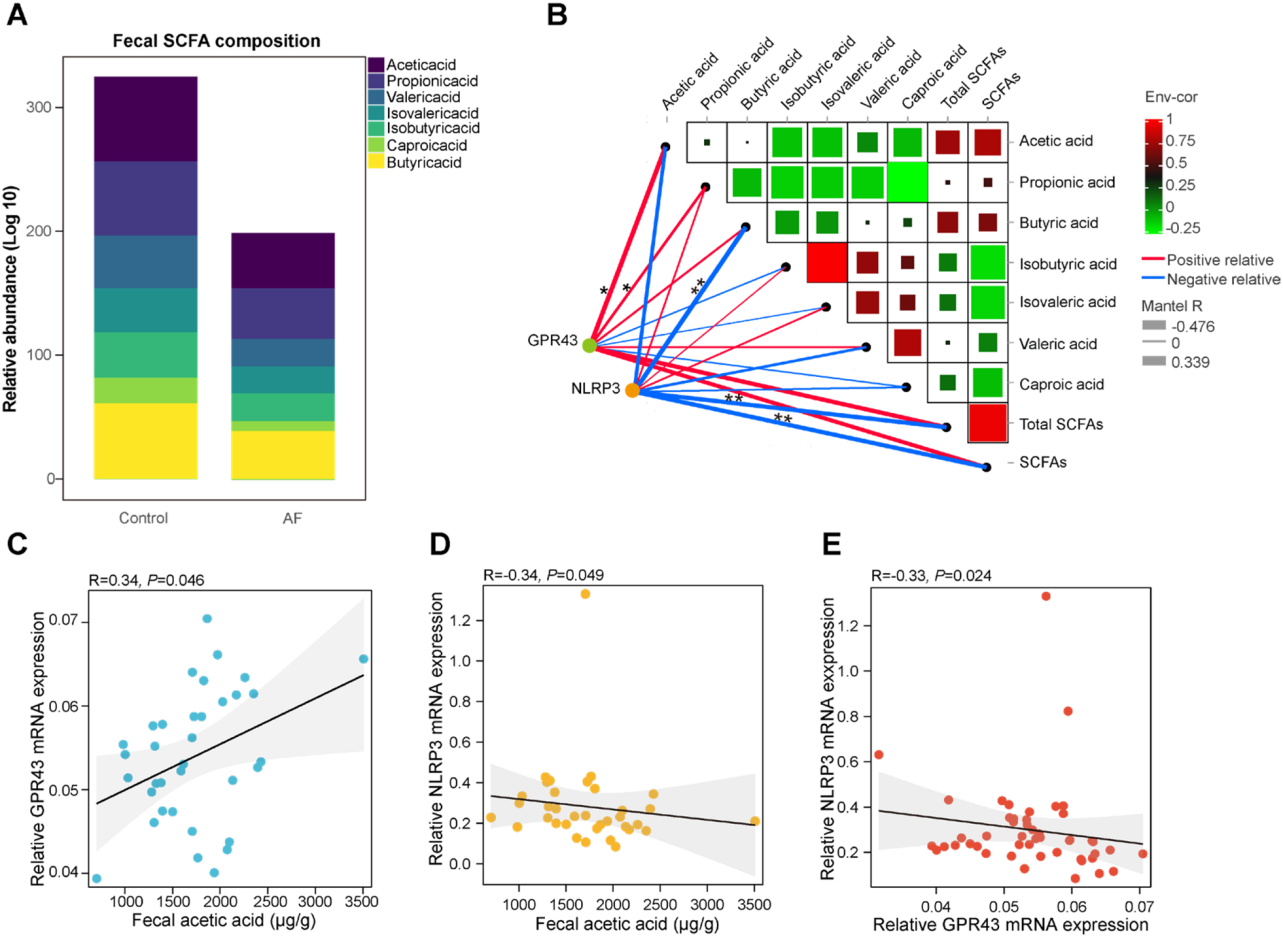
Remarkable correlation between fecal SCFAs and the levels of GPR43 and NLRP3 mRNA in leukocytes. **A** Stack bar plot depicted the relative abundance (log 10) of fecal SCFAs, including acetic acid, propionic acid, butyric acid, isobutyric acid, isovaleric acid, valeric acid and caproic acid. **B** Heatmap presented the correlation between fecal SCFAs and the network presented the correlation between leukocyte GPR43 and NLRP3 mRNA and fecal SCFAs, respectively. Total SCFAs: acetic acid, propionic acid, butyric acid, isobutyric acid, isovaleric acid, valeric acid and caproic acid; SCFAs: acetic acid, propionic acid and butyric acid. Spearman’s correlation analysis; Red, positive correlation; green, negative correlation; red line, positive correlation; blue line, negative correlation; line width, the Spearman correlation coefficient; *P < 0.05; **P < 0.01. **C**, **D** Scatter plots of correlation between acetic acid and the levels of GPR43 (*R* = 0.34, *P* = 0.046) and NLRP3 (*R* = − 0.34, *P* = 0.049) mRNA in leukocytes. **E** Significantly negative correlation between GPR43 and NLRP3 mRNA expression in leukocytes (*R* = − 0.33, *P* = 0.024). Spearman’s correlation analysis

Our previous study has demonstrated that GM-derived SCFAs (e.g., acetic acid, propionic acid and butyric acid) could protect against AF development by regulating cardiac GPR43/NLRP3 signaling [18]. In the current cohort, the levels of fecal SCFAs in 21 non-AF controls and 14 AF patients were obtained from our previous GC–MS-based dataset, including acetic acid, propionic acid, butyric acid, isobutyric acid, isovaleric acid, valeric acid and caproic acid [18]. As shown in Fig. 3A, the relative abundance of these SCFAs was lower in AF patients than in controls, while acetic acid, propionic acid and butyric acid were important components of SCFAs. To further explore the potential role of fecal SCFAs in AF patients on the expression of GPR43 and NLRP3 in peripheral blood leukocytes, Spearman’s correlation was calculated, respectively. The results revealed that GPR43 mRNA expression in leukocytes was positively related to the levels of fecal acetic acid (*R* = 0.34, *P* = 0.046) (Fig. 3B, C) and NLRP3 mRNA expression in leukocytes was negatively correlated with the levels of fecal SCFAs, especially acetic acid (*R* = − 0.34, *P* = 0.049) and butyric acid (*R* = − 0.48, *P* = 0.004) (Fig. 3B, D). Moreover, the negative correlation between the expression of GPR43 and NLRP3 mRNA in peripheral blood leukocytes was striking (*R* = − 0.33, *P* = 0.024) (Fig. 3E).

### Association between GPR43, NLRP3, and cardiac parameters

**Fig. 4:**
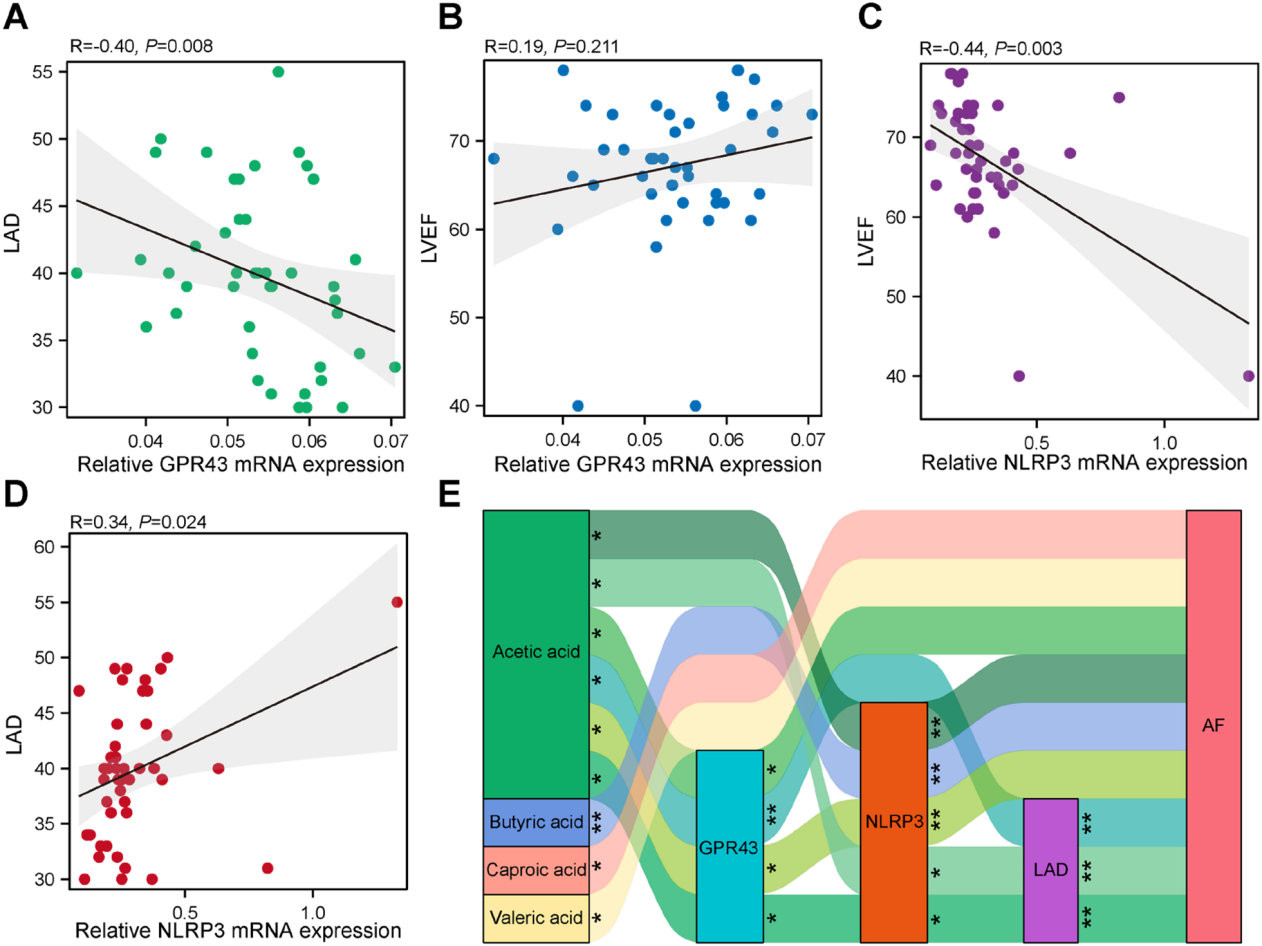
Correlation between leukocyte GPR43 and NLRP3 mRNA expression and cardiac parameters. **A**, **B** Leukocyte GPR43 mRNA expression was negatively correlated to LAD and positively correlated to LVEF. Spearman’s correlation analysis. **C**, **D** Leukocyte NLRP3 mRNA expression was positively correlated to LAD and negatively correlated to LVEF. Spearman’s correlation analysis. **E** Interrelationship of intestinal SCFAs (e.g., acetic acid, butyric acid, caproic acid and valeric acid), leukocyte GPR43 and NLRP3 mRNA expression, LAD and AF. The significant correlations using Spearman’s correlation analysis were depicted in the correlation network. *P < 0.05; **P < 0.01, ***P < 0.001

Considering that left atrial remodeling is crucial in AF progression [24], the association between GPR43, NLRP3, and left atrial diameter (LAD) was evaluated. We observed that LAD was negatively associated with GPR43 mRNA expression in leukocytes (*R* = − 0.40, *P* = 0.008) and was positively associated with NLRP3 mRNA expression (*R* = 0.34, *P* = 0.024) (Fig. 4A, D). Conversely, there was no remarkable association between right atrial diameter (RAD) and the expression of GPR43 (*R* = − 0.16, *P* = 0.302) or NLRP3 (*R* = 0.27, *P* = 0.078) in leukocytes. Left ventricular ejection fraction (LVEF) had an opposite correlation trend (*R* = 0.19, *P* = 0.211 for GPR43; and *R* = − 0.44, *P* = 0.003 for NLRP3) (Fig. 4B, C). Furthermore, a striking correlation was confirmed between AF and the expression of leukocyte GPR43 (*R* = − 0.34, *P* = 0.020) and NLRP3 mRNA (*R* = 0.48, *P* = 0.001). Then, a correlation network containing these factors with statistically significant correlation was performed and presented in Fig. 4E. AF occurrence was significantly linked to the interaction among intestinal acetic acid, GPR43, NLRP3, and LAD and was directly related to caproic acid and valeric acid. Meanwhile, NLRP3 mediated the influence of intestinal butyric acid on AF occurrence. Therefore, we speculated that the protective effect of intestinal SCFAs on AF progression is partly associated with the downregulation of leukocyte NLRP3 through activating GPR43.

### Predictive potential of GPR43 and NLRP3 in peripheral blood leukocytes for AF

**Fig. 5:**
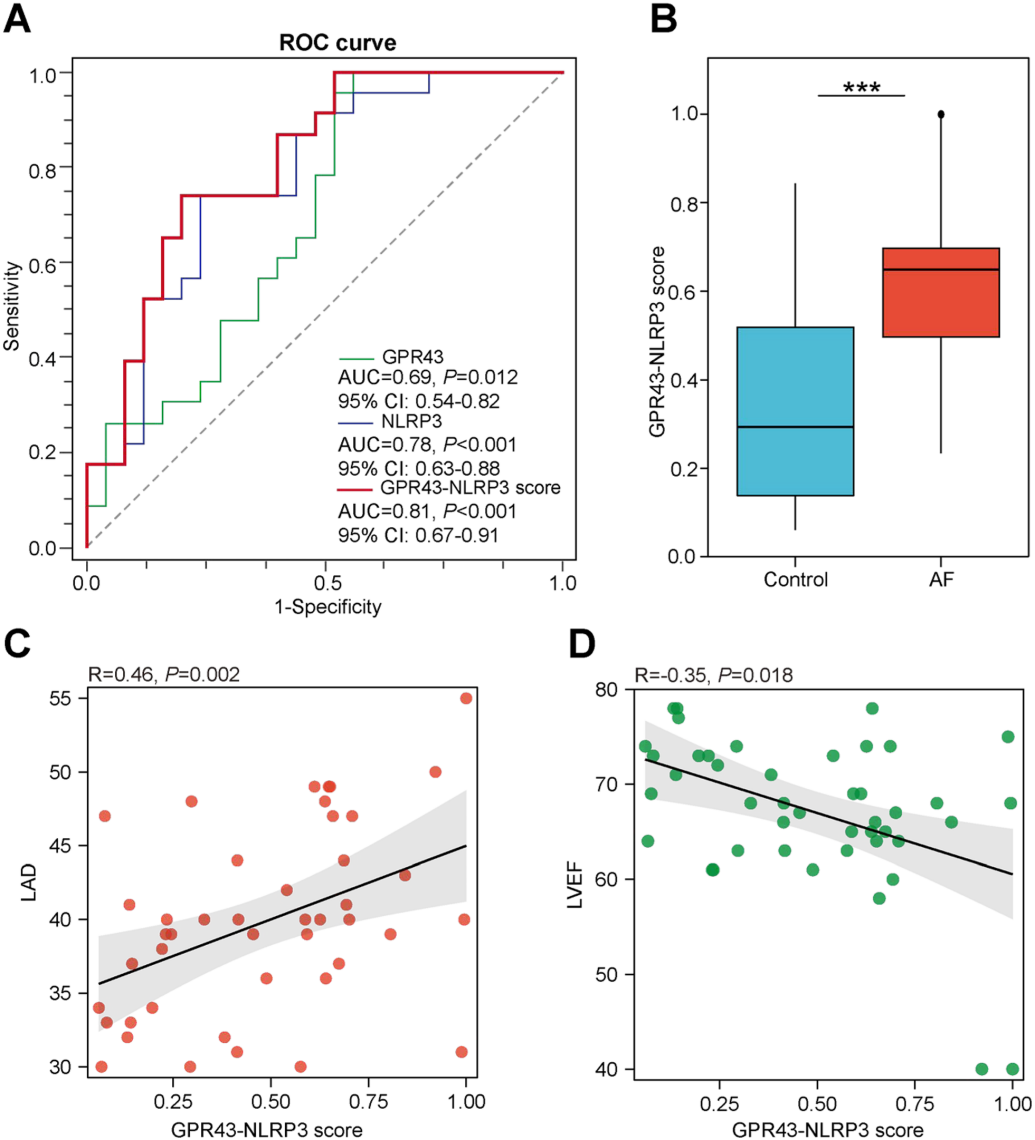
A prediction model based on leukocyte GPR43 and NLRP3 for AF. **A** Receiver operating curves (ROC) for leukocyte GPR43, NLRP3 mRNA expression and the GPR43–NLRP3 score. The area under the receiver operating curves (AUC values) were 0.69 for GPR43 (95% CI 0.54–0.82, *P* = 0.012), 0.78 for NLRP3 (95% CI 0.63–0.88, *P* < 0.001), 0.81 for the GRP43-NLRP3 score (95% CI 0.67–0.91, *P* < 0.001). **B** Higher GPR43–NLRP3 scores in AF patients than in controls. *T*-test, ***P < 0.001. **C**, **D** The GPR43–NLRP3 score was positively associated with LAD (*R* = 0.46, *P* = 0.002) and negatively associated with LVEF (*R* = − 0.35, *P* = 0.018). Spearman’s correlation analysis

Further, the analysis of ROC curves was performed to assess the predictive value of leukocyte GPR43 and NLRP3. And the results showed that the area under the receiver operating curves (AUC) of GPR43 mRNA expression was 0.69 (95% CI 0.54–0.82, *P* = 0.012) and NLRP3 mRNA expression of 0.78 (95% CI 0.63–0.88, *P* < 0.001) (Fig. 5A). Subsequently, a combined score was constructed based on logistic regression as follows: GPR43–NLRP3 score = [2.41 × (Intercept)] + [− 94.32 × GPR43] + [9.27 × NLRP3]. Compared to controls, AF patients exerted significantly higher combined scores with the higher AUC for the GPR43–NLRP3 score was 0.81 (95% CI 0.67–0.91, *P* < 0.001) (Fig. 5A, B). The AUC of age, LAD and HTN were 0.62 (95% CI 0.47–0.76, *P* = 0.161), 0.77 (95% CI 0.62–0.89, *P* < 0.001), and 0.64 (95% CI 0.49–0.77, *P* = 0.092), the recognized clinical risk factors for AF, were lower than the GPR43–NLRP3 score. Moreover, the GPR43–NLRP3 score was correlated positively with LAD (*R* = 0.46, *P* = 0.002) and negatively with LVEF (*R* = − 0.35, *P* = 0.018), which had no correlation with RAD (Fig. 5C, D). Thus, the expression of GPR43 and NLRP3 mRNA in peripheral blood leukocytes was significantly associated with AF and exerted a predictive potential for AF.

### GM-derived SCFAs may be involved in AF by regulating GPR43–NLRP3 expression

**Fig. 6:**
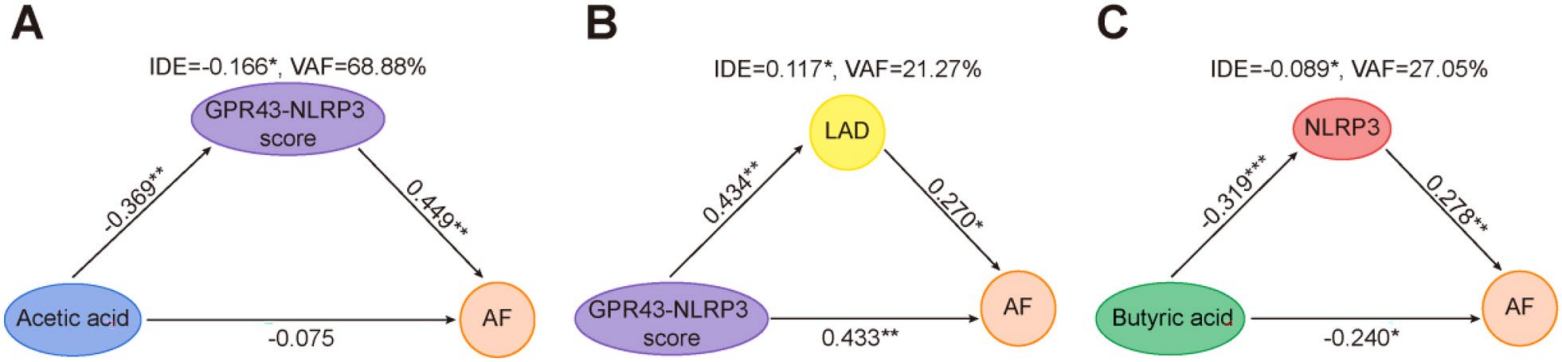
The interaction of leukocyte GPR43 and NLRP3 was involved in AF development. **A** Mediation analysis of the association between fecal acetic acid, GPR43–NLRP3 score, and AF. **B** Mediation analysis of the association between GPR43–NLRP3 score, LAD, and AF. **C** Mediation analysis of the association between butyric acid, NLRP3 and AF. Path coefficients are denoted beside each path. *IDE* indirect effect, *VAF* variance accounted for score; *P < 0.05; **P < 0.01; ***P < 0 .001

Due to the complex crosstalk between fecal SCFAs, leukocyte GPR43/NLRP3 expression, LAD and AF, PLS-SEM was applied to evaluate the mediating role of GPR43/NLRP3 in AF. Based on the variance accounted for (VAF) score, a ratio of indirect-to-total effect [25], we identified that GPR43/NLRP3 mediated 68.88% indirect effect on the effect of fecal acetic acid on AF (Fig. 6A) and indirect effect of LAD between GPR43/NLRP3 and AF was 21.27% (Fig. 6B). Moreover, intestinal butyric acid was related to AF development and the VAF for NLRP3 in peripheral blood leukocytes was 27.05% (Fig. 6C). Notably, circulating IL-1β levels were positively correlated with the GPR43–NLRP3 score (*R* = 0.33, *P* = 0.025) (Additional file 1: Fig. S1A) and negatively with butyric acid (*R* = − 0.39, *P* = 0.023) (Additional file 1: Fig. S1B). Since acetic acid and butyric acid were the most abundant constituents of intestinal SCFAs in AF patients, these results initially indicated that GM-derived SCFAs disturbances in the gut contribute to AF occurrence and may be associated with enhanced circulating inflammation mediated by altered GPR43 and NLRP3 mRNA expression in peripheral blood leukocytes, which is potential therapeutic targets for AF.

### SCFAs attenuate LPS/nigericin-induced inflammation in human THP-1 cells

**Fig. 7:**
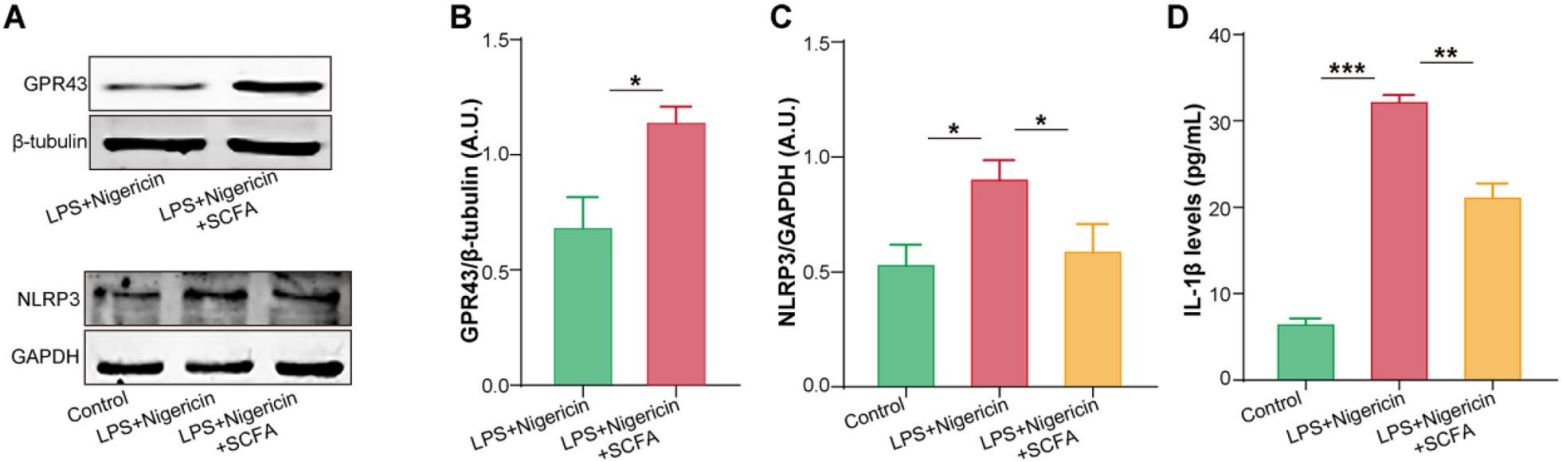
SCFAs treatment alleviated LPS/nigericin-induced inflammation in human THP-1 cells in vitro. **A**–**C** Western blotting showed SCFAs treatment significantly elevated GPR43 expression and decreased NLRP3 expression in LPS/nigericin-induced human THP-1 cells. β-Tubulin and GAPDH were utilized as endogenous control. A.U., arbitrary units; *n* = 4 for each group; *T*-test. **D** The levels of IL-1β in the culture supernatant of human THP-1 cells stimulated with or without SCFAs, LPS, and nigericin in vitro. *T*-test; *n* = 3 for each group. *P < 0.05; **P < 0.01; ***P < 0.001; data are mean ± SEM

To further determine the anti-inflammation effects of SCFAs in peripheral blood leukocytes, human THP-1 cells were stimulated with or without SCFAs (including sodium propionate, sodium butyrate, and sodium acetate), LPS, and nigericin in vitro, showing that SCFAs pre-treatment reduced the expression of NLRP3 induced by LPS/nigericin accompanied by the remarkable upregulation of GPR43 (Fig. 7A–C). Moreover, IL-1β, as a secreted protein derived from NLRP3 activation, was significantly increased in the culture supernatant of human THP-1 cells induced by LPS/nigericin, which was reversed by SCFAs treatment (Fig. 7D). These findings provided preliminary evidence for the anti-inflammation effects of SCFA/GPR43 signaling in human THP-1 cells.

## Discussion

The current study revealed significantly reduced GPR43 and elevated NLRP3 expression in peripheral blood leukocytes, coupling with higher plasma IL-1β levels in AF patients. Based on GC–MS analysis, we observed that the content of acetic acid in feces of AF patients was decreased, which was positively correlated with leukocyte GPR43 mRNA expression and negatively correlated with leukocyte NLRP3 mRNA expression. Meanwhile, a striking negative association was revealed between GPR43 and NLRP3 expression in peripheral blood leukocytes. In vitro, SCFAs treatment alleviated NLRP3 expression and IL-1β release in LPS/nigericin-induced human THP-1 cells by upregulating GPR43 expression. The interaction of GPR43/NLRP3 in leukocytes may be involved in the development of AF and has a predictive potential for AF.

GM is widely recognized as the body’s largest endocrine organ, generating biologically active metabolites that affect many physiological processes [26]. SCFAs, as the primary metabolite produced by gut microbial fermentation in the gut, have beneficial effects on inflammation, immune responses, and cardiovascular disease [27, 28]. The most important receptor of SCFAs is GPR43, widely expressed in immune cells, including monocytes, neutrophils, and eosinophils [27]. Immune cells circulate within the vasculature and are present in tissues, allowing them to rapidly detect and respond to changes in endogenous molecules and bind to relevant receptors for processing, integration and subsequent translation into effector responses to maintain cellular and tissue homeostasis [29]. Several studies suggested that the anti-inflammatory effects of SCFAs are mediated by GPR43, which is associated with leukocyte migration and cytokine secretion [30–32]. Recent clinical and animal researches have reported that decreased SCFAs is accompanied by reduced GPR43 expression, and the application of SCFAs could promote the expression of GPR43 mRNA [18, 33–36]. Thus, GPR43 expression might be regulated in an SCFA-dependent manner. Our previous studies have demonstrated disordered GM coupled with disrupted SCFAs-synthesis-related genes and decreased fecal SCFAs (e.g., acetic acid and butyric acid) in AF patients [18, 20]. The current cohort revealed that the expression of GPR43 in peripheral blood leukocytes was significantly decreased in AF patients, which was closely correlated to the reduction in fecal acetic acid levels. Patients with persistent AF had lower GPR43 mRNA expression in leukocytes than those with paroxysmal AF patients, although there was no statistical difference. Notably, unlike patients in sinus rhythm, atrial pathology in AF patients exerts lymphomonocytic infiltration [37]. Systemic inflammation is linked to AF presence and progression via pro-inflammatory cytokines, including C-reactive protein (CPR), IL-6, IL-8, TNF-α, etc*.* [38, 39]. Consistently, circulating IL-1β levels were significantly elevated in AF patients, especially psAF patients in this study. Therefore, the reduction of GM-derived SCFAs in the gut, particularly acetic acid, contributed to AF development, possibly in part associated with down-regulating leukocyte GPR43 expression, thereby increasing the release of pro-inflammatory factors.

The concomitant increase in leukocyte NLRP3 expression in this study provided further supporting evidence. The expression of NLRP3 mRNA in leukocytes significantly elevated from patients with sinus rhythm to pAF and psAF, respectively. The NLRP3 inflammasome is an intracellular supramolecular complex that mediates the activation of inflammatory mediators and is involved in many non-communicable diseases, including AF [40–42]. The activation of NLRP3, comprising a sensor molecule, the adapter apoptosis-associated speck-like protein containing a CARD complex (ASC), and the effector caspase-1, triggers the activation and release of pro-inflammatory mediators, including IL-1 family proteins and IL-18 [29]. Since the levels of ASC and caspase-1 are stable, the quantity of NLRP3 present is a crucial limiting factor in controlling its activation, and the presence of NLRP3 in myeloid cells is normally insufficient for activation [29]. Therefore, NLRP3 activation requires priming the cell by initiating transcription of the NLRP3 gene, increasing its expression, and input from G protein-coupled receptor (GPCR) signaling cascade has been confirmed to regulate this initiation step [29]. GPR43, a member of the GPCR family, has been reported to act as the SCFA downstream signaling to reduce atrial inflammatory response by suppressing NLRP3 expression and activation in the atria [18]. Similarly, this study revealed that significantly increased NLRP3 mRNA expression was negatively associated with GPR43 mRNA expression in leukocytes and fecal levels of acetic acid and butyric acid, while positively related to plasma IL-1β levels. Furthermore, SCFAs treatment reduced NLRP3 expression and IL-1β release in human THP-1 cells induced by LPS/nigericin in vitro, concomitant with the upregulation of GPR43. These results preliminarily suggested the interaction of GM-derived SCFAs and GPR43/NLRP3/IL-1β signaling in leukocytes may be involved in AF development through enhancing systemic inflammation.

Meanwhile, inflammation is involved in atrial fibrosis, particularly within the left atrium, underlying atrial structural and electrical remodeling matrix, facilitating and maintaining AF [43]. Progressive atrial dilatation is the most prominent alteration in structural remodeling and can be detected by transthoracic echocardiography [44]. In this study, LAD was positively correlated to the crosstalk between GPR43 and NLRP3 in leukocytes, which was associated with AF occurrence, and mediated 21.27% of the indirect effect. Furthermore, the combined score (GPR43–NLRP3 score) based on the levels of GPR43 and NLRP3 mRNA in peripheral blood leukocytes was constructed and had a predictive potential for AF. Thus, the present study provided initial evidence that reduced intestinal SCFAs, especially acetic acid, promoted over-expression of NLRP3 by down-regulating GPR43 on leukocytes, which in turn activated NLRP3, fostered circulating inflammation and mediated left atrial remodeling, involving in AF development. As a potential therapeutic target for AF, the specific mechanism needs further verification and exploration.

Moreover, lower SCFAs concentrations are found in circulation compared to the gut, with acetic acid, butyric acid, and propionic acid concentrations ranging 19–160, 1–13, and 1–12 µmol/L, respectively [45, 46]. Similar to most studies, the human THP-1 cells were exposed to mM-range concentrations of SCFAs in the current study, with consistent findings that SCFAs treatment decreased the LPS-induced production of inflammatory factors [47, 48]. Notably, Qi Hui et al. revealed the anti-inflammatory effect of 20 μmol/L butyrate on LPS-induced human peripheral blood mononuclear cells [49]. And SCFAs (sodium propionate, sodium butyrate, and sodium acetate) treatment at a final concentration of 200 μmol/L reduced ox-LDL-induced THP-1 cells inflammatory injury by blocking NLRP3/Caspase-1 pathway [50]. These results may suggest that physiological SCFAs concentrations in circulation are sufficient to induce an anti-inflammatory effect, with the window of efficacy remaining to be determined [51]. Another hypothesis is that short-term but repeated exposure to SCFAs at high mM concentrations as immune cells circulated through the intestinal and hepatic supply, or sustained exposure at low μM levels of SCFA, might be sufficient to influence its functions [52]. Meanwhile, the corresponding spatial mechanisms may also exist. The specific mechanism of the interaction between intestinal SCFAs, circulating immune cells, and systemic inflammation remains to be confirmed.

Notably, the protective effect of SCFA has been demonstrated in animal and cell experimental studies, and the current clinical research mainly demonstrated the correlation between SCFA and cardiovascular. However, clinical studies supporting the protective effect of SCFA are still lacking [53]. Moreover, the dramatic inter-individual variation in GM composition and host dietary habit poses significant difficulties for GM and altered metabolites converting into AF prevention and treatment targets. Individual precision treatment regiments based on machine learning datasets, including diet, GM, and genetics, as well as artificial intelligence (AI) techniques might help address this challenge [53, 54].

The current study innovatively explored the potential effects of GM-derived SCFAs on peripheral blood leukocytes, providing preliminary evidence for the protective role of SCFAs in the AF progression by regulating GPR43/NLRP3 expression and reducing IL-1β release in peripheral blood leukocytes. However, there are some limitations. Firstly, the current study was cross-sectional with a limited sample size and no strict dietary management, and lacked quantitative determination of plasma SCFAs and evaluation of GPR43/NLRP3 expression in different leukocytes. Second, further cell experiments in vitro with GPR43 knock-down need to be performed to determine its direct role in SCFAs-mediated activation of NLRP3 in leukocytes. Third, interventions with different concentration gradients are needed to explore the effective window of SCFAs. Thus, further large-scale, prospective cohort and related mechanistic studies are necessary.

## Conclusions

The expression of GPR43 in peripheral blood leukocytes was significantly decreased in AF patients, coupling with remarkably increased leukocyte NLRP3 expression and plasma IL-1β levels, which was associated with reduced levels of fecal SCFAs, particularly acetic acid. A striking negative correlation was confirmed in leukocyte GPR43 and NLRP3 mRNA expression, acting together in left atrial enlargement and AF progression. Abnormal expression of GPR43/NLRP3 in leukocytes linked to disordered GM-derived SCFAs might be involved in AF occurrence by increasing systemic inflammation and an underlying predictive and therapeutic target for AF.

### Supplementary Information

**Additional file 1: Figure S1.** Correlation between plasma IL-1β, leukocyte GPR43/NLRP3 expression, and fecal butyric acid.

## Data Availability

The datasets used and/or analyzed during the current study are available from the corresponding author on reasonable request.

## Abbreviations

AF: Atrial fibrillation
ALT: Alanine aminotransferase
AST: Aspartate aminotransferase
BMI: Body mass index
CPR: C-reactive protein
DM: Diabetes mellitus
GM: Gut microbiota
GPR43: G-protein-coupled receptor 43
GPCR: G-protein-coupled receptor
HTN: Hypertension
LAD: Left atrial diameter
LVEF: Left ventricular ejection fraction
NLRP3: NOD-like receptor family, pyrin domain containing 3
PLS-SEM: Partial least squares structural equation modeling
pAF: Paroxysmal atrial fibrillation
psAF: Persistent atrial fibrillation
qRT-PCR: Quantitative reverse transcription-polymerase chain reaction
RAD: Right atrial diameter
sCr: Serum creatinine
SCFAs: Short-chain fatty acids
TC: Total cholesterol
TG: Triglyceride
