# Supplementary material for: Disordered GPR43/NLRP3 expression in peripheral leukocytes of patients with atrial fibrillation is associated with intestinal short chain fatty acids levels
Source: Eur J Med Res. 2024 Apr 15;29:233. doi: 10.1186/s40001-024-01825-4 (PMC11017637; doi:10.1186/s40001-024-01825-4)
Supplement: Supplementary file 1 — Additional file 1: Figure S1. Correlation between plasma IL-1β, leukocyte GPR43/NLRP3 expression, and fecal butyric acid. [file 40001_2024_1825_MOESM1_ESM.docx]

**
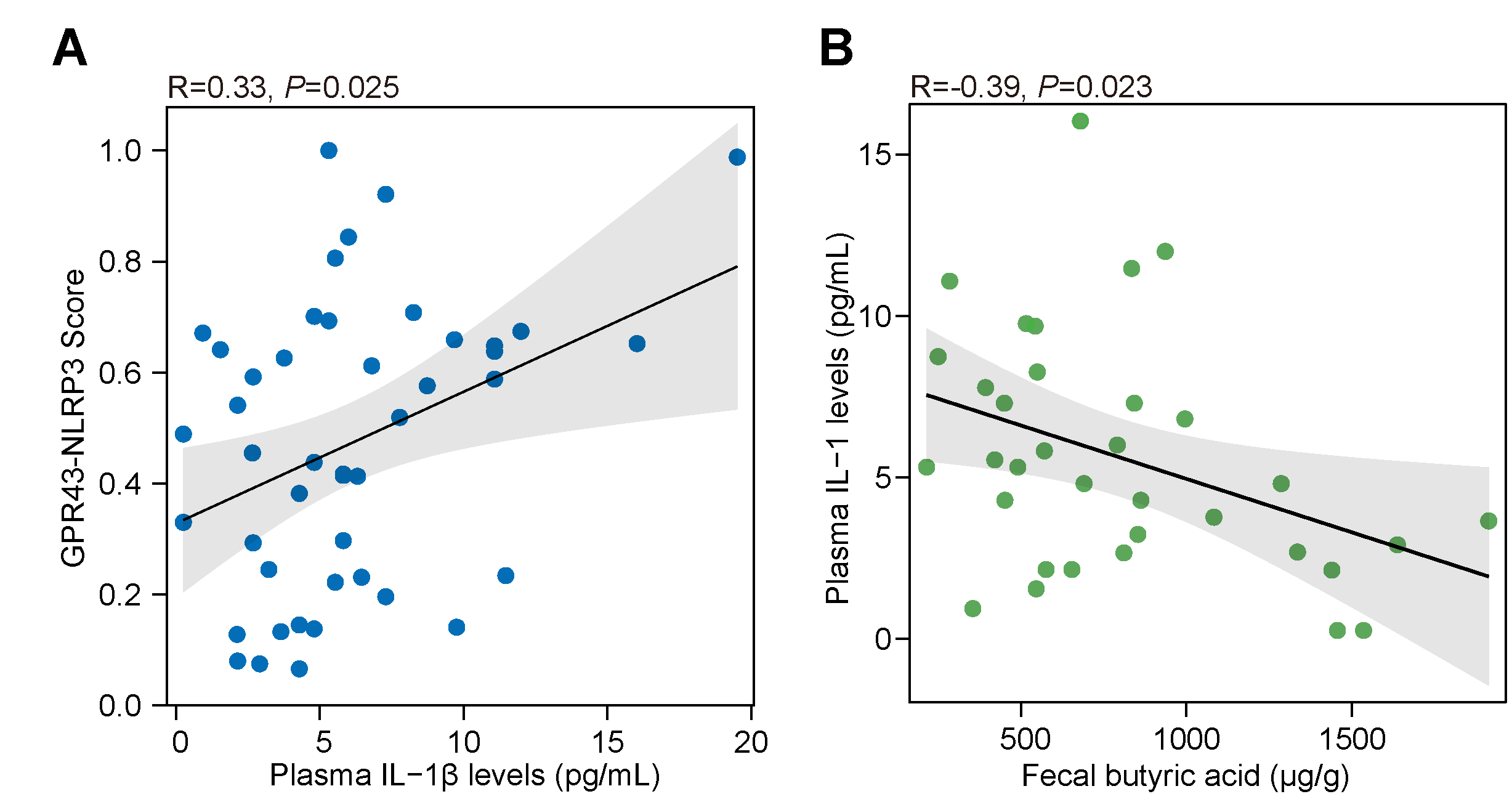
**

**Figure S1.** Correlation between plasma IL-1β, leukocyte GPR43/NLRP3 expression, and fecal butyric acid. Plasma IL-1β levels were positively associated with the GPR43-NLRP3 score **(A)** and negatively with fecal butyric acid **(B)**.
